# School bullying among Chinese third to fifth grade primary school students in a cross-sectional study: The protective effect of psychological resilience

**DOI:** 10.1371/journal.pone.0278698

**Published:** 2022-12-06

**Authors:** Liping Fei, Maoxu Liao, Lei Ke, Yanli Zou, Xin Li, Yiting Chen, Rong Zhang

**Affiliations:** 1 Department of Medical Statistics and Epidemiology, School of Public Health, Southwest Medical University, Luzhou, Sichuan Province, China; 2 Information and Education Technology Center, Southwest Medical University, Luzhou, Sichuan Province, China; 3 Department of Social Medicine, School of Public Health, Southwest Medical University, Luzhou, Sichuan Province, China; The University of Sydney, AUSTRALIA

## Abstract

School bullying is a major concern for school-aged youth and has great impacts on children’s health and well-being, and an increasing number of school bullying cases have been reported in China. Many studies have indicated that psychological resilience may have a well-established association with school bullying. However, only a limited number of studies have explored this association, especially among primary school students. The present study aimed to investigate the relationship between school bullying and psychological resilience among primary school students from a Chinese city. The participants were 6,011 primary school students aged 7–14 years who were recruited in a cross-sectional survey in Luzhou, China. The statistical significance of differences between groups was tested using the *χ*^2^ test or *t* test. Binary logistic regression analyses were conducted to explore the association between psychological resilience and school bullying. The incidence rates of bullies and victims were 30.00% (1803/6011; 95% CI: 28.84%-31.16%) and 69.89% (4201/6011; 95% CI: 68.73%-71.05%), respectively. Psychological resilience was a protective factor of school bullying among primary school students (for bullying perpetrators, *OR* = 0.76, 95%CI:0.62–0.93, and for bully victims *OR* = 0.74, 95%CI:0.61–0.90), especially among female students (for bullying perpetrators, *OR* = 0.63, 95%CI: 0.47–0.85, and for bully victims, *OR* = 0.69, 95%CI: 0.53–0.90). School bullying among primary school students in Luzhou City was highly prevalent. High levels of psychological resilience might be a protective factor in preventing primary students from being involved in school bullying, especially among females.

## Introduction

School bullying is a major concern for school-aged youth and has large impacts on children’s health and well-being. It is estimated that 1 in 2 children aged 2–17 years suffers some form of bullying each year globally [1]. A study conducted in China showed that 57.29% of junior high school students had suffered from at least one type of school bullying in the past year [2]. School bullying may cause multiple adverse effects, including physical, psychological, and other social outcomes. Physical impacts, such as injuries, bruises, and deaths caused by suicide or homicide, are easily observable [3, 4]. Regarding psychological impacts, victims and bullies are prone to suffer from depression, anxiety, and murderous ideation and behaviors [5–8]. In addition, school bullying is associated with many behavioral problems, such as smoking, substance use, high-risk sexual behaviors [9, 10] and assaulting others [11–13]. Children spend most of their day in school, so more attention should be paid to school bullying.

Previous studies have found that many factors were associated with school bullying. Boys are more likely to be involved in school bullying than girls [14, 15]. Students with quiet personalities, poor classmates’ relationships [16], poor sleep quality [17, 18], low-educated parents [14, 19], discordant family atmosphere [16, 20, 21] and low socioeconomic status families [22] were more likely to be engaged in school bullying. In contrast, students with higher levels of psychological adjustment [23], self-efficacy [24] and resilience [25] were less likely to be involved in school bullying.

Psychological resilience is the ability to withstand, adapt or recover from significant stress and adversity [26, 27]. Numerous studies have suggested that resilience plays a critical role in many fields and diseases, including as a protective factor against suicide risk [28] and as a new approach to prevent and treat posttraumatic stress disorder [29]. In particular, resilience can be a protective factor for mental health outcomes beyond adverse childhood experiences [30] and can enable people to use various resources to positively deal with crisis or stress [31].

Recent research on school bullying has mainly focused on the type of school bullying behaviors [32, 33], determinants [21], health effects and intervention strategies [34, 35], and most studies have recruited middle school students [14, 21, 35]or adults [36, 37] as subjects. Primary school students, who are in the early stage of forming behavioral habits and concepts and who experience better corrective effects on behavior and cognition, have rarely been included in existing studies. In addition, some studies have explored the relationship between resilience and school bullying, but most studies have explored this relationship indirectly [25, 38], and few studies have explored this relationship directly [23, 39]. The relationship between psychological resilience and school bullying among Chinese primary school students is not yet known. Given the gender differences in both school bullying [14, 15, 40] and psychological resilience [41, 42], we also wanted to examine whether the relationship between school bullying and psychological resilience was consistent across genders among Chinese primary school students.

The present study was designed to achieve the following four objectives: (a) to understand the current state of psychological resilience; (b) to understand the prevalence of school bullying among primary school students; (c) to explore the determinants of primary school students’ school bullying behavior; and (d) to determine whether psychological resilience has an association with school bullying behavior and whether the association is consistent across genders.

## Materials and methods

### Sample

A cross-sectional survey was conducted in Luzhou City of Sichuan Province, China. Private schools are those that are financed and constructed by nongovernmental organizations or individuals. Public schools are those that are run by state and local government agencies. Both private and public primary schools offer courses according to the curriculum plan formulated by Sichuan Province, but the number of students per class in public primary schools is approximately 1.5 times that of private primary schools. The ratio of private to public school attendance was approximately 1:5. The sampling of participants was performed using a three-stage stratified random sampling. The seven districts in Luzhou City were divided into 3 groups according to their GDP in 2018, namely high GDP districts (Jiangyang, Luxian), medium GDP districts (Longmatan, Hejiang) and low GDP districts (Naxi, Gulin and Xuyong). At the first stage, we randomly selected one district from high (Jiangyang), medium (Longmatan) and low (Naxi) districts by a lottery method. In the second stage, one private school and three public schools were randomly selected from each district using a lottery method. In the third stage, four classes were randomly selected from grades 3, 4 and 5 within each school, and all students in the selected classes were taken as the research objects. A total of 6066 students were selected as participants in this study. Fifty-five cases were excluded due to missing data on items related to school bullying and psychological resilience. Finally, the study included 6011 primary school students, consisting of 4882 (81.22%) students in public school and 1129 (18.78%) students in private school.

### Procedure

Data were collected by a self-administered questionnaire from October 2018 to January 2019. After we explained the content and significance of our research to participants’ guardians, all guardians signed informed consent forms. The response rate was 100%. Students were informed that the responses were anonymous and confidential and that there were no right or wrong answers to any of the questions. Anonymous questionnaires were conducted by trained interviewers in the absence of a teacher. The project was examined by the ethics committee of the Affiliated Hospital of Southwest Medical University (NO. KY2019128).

### Instruments

#### Sociodemographic variables and background information

Demographic variables of the participants included age, gender, grade, and character (quiet vs. general vs. outgoing). Other background information included the following: the number of good friends (≤1 vs. ≥2); member of class cadre (yes vs. no); academic performance (above average vs. average vs. below average); relationship between siblings (good vs. general vs. poor); type of school (public or private school); only child (yes vs. no); engagement in externalizing behaviors, such as smoking, playing computer games, dropping out, and wandering and not going home after school (yes vs. no); sufficient sleeping time (yes vs. no); education level of parents (university and above vs. high school vs. junior high school vs. primary school and below); parents as the main family educator (yes vs. no); parental quarrels in front of the children (yes vs. no); divorced parents (yes vs. no); and form of education after making mistakes (punishment vs. encouragement vs. both).

#### School bullying

School bullying was assessed by using a bully/victim questionnaire for primary students revised by Professor Zhang Wenxin [43]. The revised questionnaire originated from the Olweus bully/victim questionnaire for primary students [44] and was revised to be more suitable for the Chinese context. The revised questionnaire contains two sections: bullying and being bullied. Each section has seven questions that ask respondents how many times they have bullied others or been bullied in the past six months. Each question has four options, i.e., 0 times, 1–2 times, 3–4 times and 5 times and more, which are scored as 0, 1, 2 and 3 points, respectively. Bullying behavior includes (a) “scolding or making fun of others,” (b) “speaking ill of others,” (c) “deliberately kicking, pushing or hitting others,” (d) “deliberately crowding out, ostracizing or ignoring others,” (e) “giving others an unpleasant nickname due to his or her accent or dress,” (f) “forcibly taking or destroying other people’s property,” and (e) “swearing, mocking or threatening others online.” If a respondent bullied others or was bullied by others more than once in the past six months, he or she was identified as a bully or victim, respectively. A pilot survey was conducted in 2 classes to examine the students’ responses to the survey and to assess the reliability of the data collected by the questionnaire. The Cronbach’s α for the school bullying scale for the pilot sample was 0.77, and the KMO statistic was 0.76.

#### Psychological resilience

The measurement of psychological resilience was evaluated by using the Ego-Resiliency Scale (ER89) developed by Block [45]. The English version of this scale has been translated, retranslated, culturally adapted, and assessed by relevant experts [46]. There are 14 items in the scale. A Likert scale is utilized for scoring. For each item, the possible responses are 1 = “does not apply at all,” 2 = “does not apply somewhat,” 3 = “applies somewhat,” and 4 = “applies very strongly.” The 14 items are as follows: (a) “I am generous with my friends,” (b) “I quickly get over and recover from being startled,” (c) “I enjoy dealing with new and unusual situations,” (d) “I usually succeed in making a favorable impression on people,” (e) “I enjoy trying new foods I have never tasted before,” (f) “I am regarded as a very energetic person,” (g) “I like to take different paths to familiar places,” (h) “I am more curious than most people,” (i) “Most of the people I meet are likeable,” (j) “I usually think carefully about something before acting,” (k) “I like to do new and different things,” (l) “My daily life is full of things that keep me interested,” (m) “I would be willing to describe myself as a pretty ‘strong’ personality,” and (n) “I get over my anger at someone reasonably quickly.” The scale has a total score of 56 points. The higher the score, the better the psychological resilience. In the pilot sample, the Cronbach’s α for the psychological resilience scale was 0.76, and the KMO statistic was 0.87. In this study, psychological resilience was divided into three levels based on participants’ scores: low level, with 37 points and below; medium level, with 38–44 points; and high level, with 45 points and above. To determine these cutoff points for the low, medium and high levels, the psychological resilience scores of the subjects were sorted in ascending order and then divided into thirds based on the total number of students. Scores below one-third of the total number of people belonged to the low level, scores higher than two-thirds of the total number of people belonged to the high level, and those between one-third and two-thirds were the medium level.

#### Bullying awareness

Referring to previous literature, school bullying awareness was assessed by a self-made questionnaire. The scale includes 17 items on physical bullying, verbal bullying and cyber bullying. The items are scored on a Likert scale where 1 = “correct,” 2 = “slightly wrong,” 3 = “wrong,” and 4 = “strongly wrong.” The scale has a total score of 68 points. The higher the number of points, the better the awareness of school bullying. In the pilot sample, the Cronbach’s α for the school bullying awareness scale was 0.83, and the KMO statistic was 0.94.

### Data analysis

We used EpiData Version 3.1 software (EpiData Association., DNK) and double entry to build the database. SPSS Statistics Version 25.0 (IBM Inc., Amonk, NY) was used for data analysis. Numerical data are shown as means ± standard deviations, and count data are displayed as proportions or ratios. The *χ*^2^ test was used to examine differences in respondents’ experience of school bullying or being bullied. The linear relationship between the level of psychological resilience and school bullying was analyzed using the Mantel-Haenszel trend test. Logistic regression was used to examine the influence of demographic characteristics, psychological resilience, and school bullying awareness on adolescents’ bullying or victimization experience. Statistical significance was defined as *p <* 0.05.

## Results

### Socio-demographic characteristics of participants

As is reported in Table 1, the average age of the students was 9.53±0.97 years. Approximately 80% of the students were enrolled in public school. More than 90% of the students had 2 or more friends in class. Members of class cadres accounted for 36.08% of the students. The rates of respondents who reported that their academic performance was above average, average and below average were 32.85%, 56.06% and 10.23%, respectively. A total of 88.77% of the respondents reported that their relationship with siblings was good. Approximately half (57.63%) and two-thirds (67.49%) of the students had sufficient sleeping time and had bad behavior, respectively. One-third (29.57%) of the students reported that their parents quarreled in front of them. A total of 12.19% of the students reported that their parents had divorced. Approximately half of the children adopted a positive form of education after making mistakes. The mean psychological resilience score was 40.00±8.09, ranging from the middle to lower levels.

**Table 1:** Characteristics of the participants from Luzhou City, China n(%)/Mean±SD.

Characteristics	n(%)/Mean±SD SDSD(SD)	Characteristics	n(%)/Mean±SD
Enrolled school		Externalizing behavior*	
Public	4,882(81.22)	Yes	3,953(67.49)
Private	1,129(18.78)	No	1,904(32.51)
District		Sufficient sleeping time*	
Jiangyang	3,455(33.44)	Yes	3,432(57.63)
Longmatan	4,016(38.87)	No	2,523(42.37)
Naxi	2,860(27.69)	Father’s education level*	
Gender		College and above	1,657(32.73)
Male	3,109(51.75)	High school	1,560(30.82)
Female	2,818(46.90)	Middle school	1,254(24.77)
Grade		Primary school or below	591(11.68)
3	1,806(30.04)	Mother’s education level*	
4	2,108(35.07)	College and above	1,549(30.38)
5	2,097(34.89)	High school	1,579(30.97)
Character		Middle school	1,247(24.46)
Quiet	826(13.74)	Primary school or below	724(14.20)
General	2,216(36.87)	Parents as the main family educator *	
Outgoing	2,908(48.38)	Yes	4,780(81.09)
Number of good friends		No	1,115(18.91)
≤1	359(5.97)	Parents quarreled in front of children*	
≥2	5,597(93.11)	Yes	1,751(29.57)
Class cadre member		No	4,171(70.43)
Yes	2,145(36.08)	Divorced parents*	
No	3,800(63.92)	Yes	731(12.19)
Academic performance		No	5,264(87.81)
Above average	1,974(32.85)	Form of education*	
Average	3,369(56.06)	Punishment	963(16.51)
Below average	615(10.23)	Both	1,564(26.81)
Relationship between siblings		Encouragement	3,306(56.68)
Good	3,629(88.77)	Psychological resilience	
General	359(8.78)	High	2,277(37.88)
Poor	100(2.45)	Middle	1,982(32.97)
Age	9.53±0.97	Low	1,752(29.15)
Recognition score	40.00±8.09	Psychological resilience	40.00±8.09

### Occurrence of different kinds of school bullying

In the last 6 months, a total of 30.00% (1803/6011; 95% CI: 28.84%-31.16%) of primary school students reported participating in bullying others in different ways, and 69.89% (4201/6011; 95% CI: 68.73%-71.05%) reported experiencing various kinds of school bullying. The main bullying behaviors included “scolding or making fun of others,” “speaking ill of others” and “deliberately kicking, pushing or hitting others.” The main bullying behaviors that victims had experienced were “deliberately kicking, pushing or hitting others,” “speaking ill of others” and “scolding or making fun of others” (Table 2).

**Table 2:** Occurrence of different kinds of bullying behavior in primary schools in Luzhou City (n (%)).

Num.	Bullying behavior	Bullies	Victims
1	Scolding or making fun of others	916(15.24)	2,499(41.57)
2	Speaking ill of others	740(12.31)	2,628(43.72)
3	Deliberately kicking, pushing or hitting others	708(11.78)	2,677(44.54)
4	Deliberately crowding out, ostracizing or ignoring others	383(6.37)	1,332(22.17)
5	Giving others an unpleasant nickname due to his or her accent or dress	360(5.99)	1,087(18.09)
6	Forcibly taking or destroying other people’s property	196(3.26)	1,074(17.87)
7	Swearing, mocking or threatening others online	125(2.08)	307(5.11)
Total		1,803(30.00)	4,201(69.89)

### Characteristics and frequency distribution for bullies and victims

As reported in Table 3, the incidence rate of being bullied among boys (2,265/3,109, 72.85%) was significantly higher than that of girls (1,877/2,818, 66.61%). Similarly, the incidence rate of bullying among boys (1,101/3,109, 35.41%) was also significantly higher than that of girls (674/2,818, 23.92%).

**Table 3:** Characteristics and frequency distribution for bullies and victims (n (%)).

Variable	Bullies	Victims
Enrolled school		
Public	1,528(31.30)**	3,557(72.86)**
Private	275(24.36)	644(57.00)
Gender		
Male	1,101(35.41)**	2,265(72.85)**
Female	674(23.92)	1,877(66.61)
Grade		
3	558(30.90)	1,346(74.53)**
4	599(28.42)	1,456(69.07)
5	646(30.81)	1,399(66.71)
Character		
Quiet	231(27.97)**	575(69.61)**
General	797(35.97)	1,676(75.63)
Outgoing	741(25.48)	1,901(65.37)
Number of good friends		
≤1	145(40.39)**	301(83.84)**
≥2	1,635(29.21)	3,853(68.84)
Class cadre member		
Yes	498(23.22)**	1,365(63.64)**
No	1,283(33.76)	2,786(73.32)
Academic performance		
Above average	469(23.76)**	1,241(62.87)**
Average	1,043(30.96)	2,424(71.95)
Below average	269(43.74)	498(80.98)
Externalizing behavior		
Yes	1,435(36.30)**	3,065(77.54)**
No	308(16.18)	1,018(53.47)
Sufficient sleeping time		
Yes	977(28.47)*	2,308(67.25)**
No	804(31.87)	1,850(73.33)
Relationship between siblings		
Good	1,001(27.58)**	2,467(67.98)**
General	150(41.78)	295(82.17)
Poor	48(48.00)	92(92.00)
Father’s education level		
College and above	426(25.71)**	1,077(65.00)**
High school	448(28.72)	1,078(69.10)
Middle school	396(31.58)	901(71.85)
Primary school or below	225(38.07)	459(77.66)
Mother’s education level		
College and above	383(24.73)**	1,008(65.07)**
High school	452(28.63)	1,066(67.51)
Middle school	397(31.84)	896(71.85)
Primary school or below	259(35.77)	562(77.62)
Parents as the main family educator		
Yes	1,366(28.58)**	3,260(68.2)**
No	388(34.80)	851(76.32)
Parents quarreled in front of children		
Yes	752(42.95)**	1,486(84.87)**
No	1,015(24.33)	2,645(63.41)
Divorced parents		
Yes	1,517(28.82)**	3,611(68.60)**
No	276(37.76)	575(78.66)
Form of education		
Punishment	408(42.37)**	809(84.01)**
Both	566(36.19)	1,210(77.37)
Encouragement	763(23.08)	2,061(62.34)
Recognition score	61.72 ±7.82**	63.02± 6.79**
Psychological resilience		
Low	855(37.55)**	1,772(77.82)**
Middle	592(29.87)	1,391(70.18)
High	356(20.32)	1,038(59.25)

Note.

* *p* < 0.05;

** *p* < 0.001.

The test level is *α* = 0.05

In addition, the rates differed among those with different levels of psychological resilience. As for being bullied, the incidence rates of students with high, medium and low levels of psychological resilience were 1,772/2,277 (77.82%), 1,391/1,982 (70.18%) and 1,038/1,752 (59.25%) respectively. As for bullying, the incidence rates of students with high, medium and low levels of psychological resilience were 855/2,277 (37.55%), 592/1,982 (29.87%) and 356/1,752 (20.32%) respectively. Interestingly, rates of bullying and being bullied decreased as the level of psychological resilience increased (for being bullied, *χ*^2^_*trend*_ = 162.46, *p* < 0.001; for bullying, *χ*^2^_*trend*_ = 140.01, *p* < 0.001).

In addition, students who were not members of class cadres, had poor academic performance, no more than one good friend, bad relationships with siblings, insufficient sleeping time and bad behavior reported a higher incidence rate of bullying or being bullied than other students (*p* < 0.001). Students whose parents quarreled in front of them, whose parents were divorced, and who were educated in a negative way after making mistakes had a higher rate of bullying or being bullied (*p* < 0.001) (Table 3).

### Relationship between psychological resilience and school bullying by gender

After controlling for confounding factors, we found that students with high psychological resilience were less likely to be involved in bullying others and being bullied (*OR* = 0.76, 95% CI: 0.62–0.93, *OR* = 0.74, 95% CI: 0.61–0.90, respectively). After stratified analysis by gender, the above relationship was only found among the female student samples (*OR* = 0.63, 95% CI: 0.47–0.85, *OR* = 0.69, 95% CI: 0.53–0.90, respectively). Students who were not members of class cadres, had poor academic performance, had parents who quarreled in front of them, had externalizing behavior, had insufficient sleeping time, had lower recognition scores, had divorced parents and were punished after making mistakes were at a higher risk for bullying others. Students who were in public school, had poor academic performance, had parents who quarreled in front of them, had no more than one good friend, had externalizing behavior, had bad relationships between siblings, had insufficient sleeping time, had lower recognition scores, had divorced parents and were punished after making mistakes were at a higher risk for bullying others (Table 4).

**Table 4:** Binary logistic regression of school bullying (OR (95% CI)).

Variables	Bullies			Victims		
	Male	Female	total	Male	Female	total
Private school (ref: public)	-	-	-	0.67* (0.52–0.88)	0.58** (0.44–0.77)	0.65** (0.54–0.78)
Class cadre member (ref: no)	0.75* (0.58–0.97)	0.76* (0.60–0.97)	0.77* (0.64–0.92)	-	-	-
Academic performance (ref: above average)						
Average	1.24 (0.97–1.58)	-	1.20 (1.00–1.43)	1.38* (1.08–1.77)	1.19 (0.95–1.50)	1.26* (1.06–1.48)
Below average	1.65* (1.14–2.38)	-	1.41* (1.06–1.88)	1.35 (0.88–2.08)	1.97* (1.22–3.19)	1.49* (1.08–2.05)
Parents quarreled in front of children (ref: no)	1.91** (1.52–2.38)	1.62** (1.27–2.08)	1.77** (1.51–2.09)	2.21** (1.65–2.95)	1.90** (1.45–2.49)	2.07** (1.70–2.52)
≥2good friends (ref:≤1)	-	-	-	0.45* (0.23–0.86)	0.35* (0.19–0.68)	0.41** (0.26–0.64)
Bad behavior (ref: no)	2.23** (1.71–2.91)	2.32** (1.79–3.01)	2.32** (1.93–2.79)	2.77** (2.17–3.52)	2.14** (1.73–2.64)	2.40** (2.05–2.80)
Relationship between siblings (ref: good)						
General	-	-	-	-	1.21 (0.78–1.88)	1.23 (0.89–1.70)
Poor	-	-	-	-	5.54* (1.27–24.12)	4.23* (1.65–10.86)
Sufficient sleeping time (ref: no)	-	0.78* (0.62–0.98)	0.85* (0.73–1.00)	0.77* (0.61–0.98)	0.73* (0.59–0.90)	0.77* (0.65–0.9)
Recognition score	0.95** (0.94–0.97)	0.96** (0.94–0.98)	0.96** (0.94–0.97)	0.97* (0.95–0.99)	0.98* (0.96–0.99)	0.97** (0.96–0.99)
Divorced parents (ref: no)	-	1.44* (1.01–2.06)	1.40* (1.10–1.79)	1.63* (1.06–2.50)	1.68* (1.12–2.51)	1.71** (1.28–2.28)
Form of education (ref: punishment)						
Both	1.04 (0.77–1.4)	0.67* (0.48–0.95)	0.85 (0.68–1.06)	0.78 (0.53–1.17)	1.04 (0.70–1.56)	0.88 (0.66–1.16)
Encouragement	0.59** (0.44–0.77)	0.56** (0.4–0.77)	0.57** (0.46–0.71)	0.44** (0.31–0.63)	0.69* (0.48–0.99)	0.54** (0.42–0.70)
Psychological resilience (ref: low)						
Middle	-	0.99 (0.76–1.29)	0.92 (0.77–1.1)	-	0.97 (0.75–1.27)	0.89 (0.74–1.08)
High	-	0.63* (0.47–0.85)	0.76* (0.62–0.93)	-	0.69* (0.53–0.90)	0.74* (0.61–0.90)

Note. Confounding factors adjusted for in total model included type of scool, gender, character, number of good friends, class cadre member, academic performance, externalizing behavior, sufficient sleep time, relationship between siblings, father’s education level, mother’s education level, parents quarreling in front of the children, divorced parents, form of education, and recognition score. In the male and female models, other confounding factors in the total model other than gender were adjusted.

* *p* < 0.05;

** *p* < 0.001.

The test level is *α* = 0.05

## Discussion

The primary school period is crucial to the formation of children’s thoughts and behaviors, which will influence their behavior patterns in middle school and even in adulthood. A negative association between psychological resilience and school bullying was found among female primary school students in this study. The findings of this study may provide new perspectives and strategies for the intervention of school bullying.

### Psychological resilience in primary school students

The findings on the psychological resilience of primary school students were not optimistic. The psychological resilience of pupils in the present study was at the middle and lower levels (average score of 40.00±8.09), which is lower than that in similar studies conducted in China [47, 48]. Previous research has shown that psychological resilience may be related to the family environment, parenting style, parents’ educational level and the attention educators pay to students [49]. The psychological resilience of primary school students is still at an immature stage and is highly malleable. Psychological development at this stage plays an extremely important role in students’ future growth. Therefore, parents, schools and societies should focus on cultivating the psychological resilience of primary school students, starting by creating a happy and harmonious family atmosphere and a friendly school atmosphere between teachers and students and by promoting the positive development of primary school students’ psychological resilience.

### The prevalence of school bullying

The present study found that school bullying was frequently observed among primary school students (grades 3 to 5), with victimization (69.89%) being more prevalent than perpetration (30.00%). In addition, the prevalence of school bullying in this study was much higher than that in Portugal [17] and Japan [50]. Our results were also inconsistent with other studies conducted in other cities of China. More specifically, the prevalence in our study was lower than the prevalence reported in studies conducted in Changsha [51], Shenzhen Bao’an District [52] and Henan Province [53]. In contrast, it was much higher than those reported in studies carried out in Guizhou and Anhui Provinces [54], 11 provinces or autonomous regions in China [15] and urban China [55]. There are several possible explanations. First, school bullying has recently become a national concern in China. More light has been shed on this problem, so some policies and measures have been implemented by the administration, schools and parents to prevent it. Second, the participants in the studies were slightly different in terms of factors such as area and age. Finally, the definition of school bullying can vary slightly, which may lead to inconsistent findings.

### Determinants of school bullying

Similar to other studies [15, 56], we found a number of factors related to school bullying. In this study, the protective factors for being involved in school bullying were sufficient sleeping time, good academic performance, higher recognition scores, and good relationships with siblings. Being punished after making mistakes, having externalizing behaviors, having divorced parents and parents who quarreled in front of children were risk factors for involvement in school bullying. Previous studies have shown that there is a close relationship between sleep and involvement in bullying situations [57]. Good academic performance was a protective factor against school bullying, which was consistent with Galal’s finding [58]. Engaging in behaviors such as smoking, truancy, playing computer games or mobile games and wandering outside after school was a risk factor for school bullying [15, 59]. An earlier study also demonstrated that poor family atmosphere, physical punishment were closely related to school bullying [60], which was consistent with our finding.

### Association between school bullying and psychological resilience

Psychological resilience was closely associated with school bullying, especially among primary school girls. Previous studies have shown significant differences in psychological resilience between boys and girls [61, 62]. There were also differences in the prevalence of school bullying between males and females, which was consistent with several studies reporting that males are the main participants in school bullying [15, 63]. Therefore, we analyzed school bullying in male, female and overall respondents. Our study found that a high level of psychological resilience was a protective factor against being involved school bullying, either as a victim or bully, among all students. This finding was almost consistent with a previous study, and we further found that this relationship existed among female students. Cohen et al. [39] revealed that in primary school in Israel, individual resilience was a significant protective factor against peer bullying and victimization. However, whether there were differences across genders was not mentioned. The results of our study suggested that psychological resilience was a protective factor against school bullying among female students but not among males. As one explanation, previous studies in China showed that females are more psychologically resilient than males [41, 64]. Since resilience refers to the ability to recover from significant stress and adversity, individuals with high resilience can solve problems through positive behavior, while people with low resilience mostly protect themselves through violence [65, 66]. Therefore, this protective relationship between psychological resilience and bullying in school can also be seen in the general population, especially among female primary school students. Our findings suggested that improving students’ mental resilience may help prevent and control bullying in schools, at least among female students. In addition, future research should also focus on how well students’ parents understand these issues and their underlying psychology, concerns, and actions. These are important for planning prevention strategies involving parents.

There are some limitations to this study. First, the study used a cross-sectional design. Given the nature of the cross-sectional design, the causality between resilience and school bullying cannot be established in this study. Longitudinal studies should be used to further understand the causality between psychological resilience and school bullying in future research. Second, the subject of this study was a large sample of primary school students from Luzhou City, Sichuan Province, which is a typical third-tier city in China. In view of the different socioeconomic conditions between different cities, the generalizability of the findings to populations in other cities, especially those with other socioeconomic conditions, is limited. Third, given that school bullying is a sensitive issue, all the measures in this study were self-reported. Although the purpose and significance of this research was told to the participants before the survey and questionnaires were completed independently and anonymously to minimize bias, there may still have been a gap between the participants’ reports and reality. More empirical research is needed to replicate our findings. Despite these limitations, this study provided a new perspective for reducing school bullying. Education administrators can encourage schools to offer mental health classes, adversity training, etc. to improve students’ psychological quality and psychological resilience in order to reduce school bullying. Parents are also encouraged to communicate more with their children to create a good family atmosphere and participate in reducing bullying in schools.

## Conclusions

The results of this study indicated that school bullying is highly prevalent among primary school students in Luzhou City. A high level of psychological resilience might be a protective factor in preventing primary students from school bullying, especially among females. Some new directions for future research are indicated in the present study. Further research on children’s school bullying should include the elements of psychological resilience and examine differences and similarities between genders. In addition, future longitudinal studies on the school bullying behavior of pupils need to further verify the relationship between psychological resilience and school bullying, as the specific pathway of this influence is not yet clear.

## Supporting information

S1 File School bullying. (XLS)
